# 525 Dysphagia in Thermal Injury: The Impact of Inhalation Injury on Incidence and Recovery

**DOI:** 10.1093/jbcr/irac012.155

**Published:** 2022-03-23

**Authors:** Bailey Weiner, Harper Halfacre, Jenny Lee, John A Harvin

**Affiliations:** Memorial Hermann Texas Medical Center, Houston, Texas; Memorial Hermann Texas Medical Center, Houston, Texas; Memorial Hermann Texas Medical Center, Houston, Texas; Memorial Hermann Texas Medical Center, Houston, Texas

## Abstract

**Introduction:**

Dysphagia is known to be a prevalent condition in the burn population. Dysphagia can lead to adverse events such as aspiration pneumonia, dehydration, and malnutrition. However, there is little data on the incidence and duration of dysphagia specifically in the inhalation injury subset of burn patients. The aim of this study is to determine the incidence and factors that contribute to dysphagia in the inhalation injury burn population as compared to the cutaneous burn population.

**Methods:**

A retrospective study was conducted of patients admitted to a burn center from January 2016 - January 2021 and intubated for >48 hours. Patients who died during hospitalization or transferred hospitals were excluded. Dysphagia duration was analyzed based on days free from the ventilator. Two groups were compared: 1) non-inhalational injury vs inhalational injury patients and 2) Grade 1 vs Grade ≥2 inhalational injury patients. Statistical analysis included student’s t-test, chi-square test, and Kruskal-Wallis test. Bayesian generalized linear models were created to measure the independent association of inhalational injuries with the outcomes.

**Results:**

During the study period, 142 patients were admitted, of whom 49 patients had inhalation injury (35%). Inhalational injury patients had a lower %TBSA burn than non-inhalational injury patients (mean 18% ± 21% vs 31% ± 15%, p< 0.001). There were no significant differences in age, sex, tracheostomy placement, ventilator days, or hospital length of stay between the two groups. The inhalational injury group had a higher rate of dysphagia at the first Speech Language Pathologist (SLP) instrumental assessment (88% versus 51%, p< 0.001) and at discharge (55% versus 28%, p=0.001). After controlling for %TBSA, inhalational injury was independently associated with an increased odds of dysphagia at first SLP instrumental assessment (OR 13.0, 95% CrI 4.7-43.4, posterior probability ≥99%) and at discharge (OR 3.2, 95% CrI 1.5-6.9, posterior probability ≥99%). Additionally, inhalational injury patients had a longer period of dysphagia post-extubation with an average of 9.5 vs 7.1 days to any diet (p< 0.006) and average of 12.6 vs 7 days to a regular texture diet (p < 0.001). Grade 1 inhalational injuries had no difference in duration of dysphagia or dysphagia at discharge compared to Grade ≥2 inhalational injury patients.

**Conclusions:**

Inhalational injury was independently associated with dysphagia upon initial SLP instrumental assessment and at discharge. Patients with an inhalational injury also had a longer dysphagia resolution time. The severity of the inhalation injury did not impact dysphagia incidence.

